# How do Australian social media users experience self-harm and suicide-related content? A National cross-sectional survey comparing young people and adults

**DOI:** 10.1186/s12889-025-25646-0

**Published:** 2025-12-04

**Authors:** Jo Robinson, Louise La Sala, Bridget Kenny, Charlie Cooper, Michelle Lamblin, Matthew Spittal, Caroline Gao, Marina Kunin, Angela Nicholas, Atria Rezwan, Maddox Gifford, Jane Pirkis, Ann John

**Affiliations:** 1 Orygen, 35 Poplar Road, Parkville, 3052 Australia; 2 Centre for Youth Mental Health, University of Melbourne, Grattan Street, Parkville, 3010 Australia; 3 Centre for Mental Health, Melbourne School of Population and Global Health, University of Melbourne, Grattan Street, Parkville, Australia; 4 Swansea University Medical School, Swansea University, Singleton Park, Swansea, SA2 800 Wales, U.K.

**Keywords:** Social media, Suicide, Self-harm, Suicide prevention, Online safety, National survey

## Abstract

**Background:**

Rates of self-harm and suicide appear to be increasing in young people and many attribute this to social media use. However, high quality studies examining young people’s experiences of self-harm and suicide-related content on social media, and the impact on wellbeing, are lacking.

**Methods:**

An online national cross-sectional survey was conducted between January and March 2024. Quota sampling was used. Participants from across Australia were recruited from the Roy Morgan Single Source Panel, a panel managed by Pureprofile and via snowball sampling. Descriptive statistics were used to examine respondents’ experiences; logistic regressions examined differences between young people and adults.

**Results:**

Three thousand five hundred forty-nine individuals (895 young people; 2,654 adults) completed the survey. Just over half had been exposed to self-harm or suicide-related content on social media. Young people were more likely to be exposed than adults (Adjusted OR 3.81; 95%CI: 3.18–4.58). For most people exposure worsened their mood and a minority reported engaging in self-harm as a result; again this was more common in young people (Adjusted OR 4.02, 95%CI: 2.42–6.86). However, many people, in particular young people, reported using social media to seek support.

**Conclusion:**

There is concern about the impact of social media on self-harm and suicide and our findings support the need for improvements to online safety. However, the fact that people use social media to access help suggests that a nuanced and evidence-based approach is required that includes the perspectives of young people and those with lived experience.

**Supplementary Information:**

The online version contains supplementary material available at 10.1186/s12889-025-25646-0.

## Introduction

Suicide is the leading cause of death among Australian young people [1] and the fourth leading cause worldwide [2]. Self-harm is more common and is a key indicator of future suicide [3, 4]. Self-harm is defined as an act in which a person injures or poisons themselves with a motive that may or may not involve the intention of ending their life and survives [5]. Research has shown an increase in hospital presentations and admissions for self-harm, primarily driven by young women and girls aged 13–17, both in Australia [6, 7] and elsewhere [8].

The reasons for suicide and self-harm are complex, however many have cited the rise in social media use as a possible contributor [9]. For example, there are concerns that exposure to certain types of content, such as graphic depictions of suicide or self-harm may contribute to psychological distress and increase an individual’s likelihood of engaging in similar behaviour [10]. However, social media is popular among young people and studies have also identified benefits, such as the ability to access support and to communicate with others experiencing similar difficulties in an accessible way [11]. This has led some authors to conclude that social media use is neither entirely harmful, nor entirely helpful when it comes to self-harm or suicide [11], rather it is complex and nuanced, and its impact can vary from person to person and moment to moment [12].

However, robust population-level studies are lacking [11]. Thus, there is a need for high-quality studies that can quantify the extent to which young people (aged 25 and below), compared to adults, use social media to communicate about self-harm and suicide, the extent to which they are exposed to self-harm and suicide-related content on social media and the impact this has on them. This is relevant because although young people tend to use social media more frequently [12], it is invariably adults who make decisions regarding online safety, including the safety measures implemented by many social media companies. Such measures include community guidelines and features that help restrict or control the availability or visibility of certain types of content. For example, on Meta platforms (e.g., Instagram and Facebook), graphic images of self-harm violate community guidelines and are either removed or blurred to limit visibility. However, to date, few studies have examined how helpful these strategies are for users.

One exception was a national survey of a convenience sample of 5,294 individuals commissioned by The Samaritans in the United Kingdom (UK) (https://media.samaritans.org/documents/Samaritans_How_social_media_users_experience_self-harm_and_suicide_content_WEB_v3.pdf). In this study over three quarters of respondents reported having viewed self-harm-related content by age 14 or younger and 83% reported being exposed to this content without actively searching for it (e.g., via TikTok’s ‘For you’ function). When asked about the utility of the platforms’ safety features, responses were mixed. For example, whilst almost all respondents had seen a post that had been censored or blurred, most said they would click on it anyway.

A second study, conducted in Australia, sought consensus between young people and suicide prevention professionals on the steps they thought social media companies should take to improve platform safety in relation to self-harm and suicide [13]. There was general consensus that self-harm- and suicide-related content should be restricted and that safety policies should cover content that promotes self-harm or suicide, graphic imagery, as well as suicide games, pacts and hoaxes [13].

The limited data quantifying the extent to which young people, compared to adults, experience self-harm- and suicide-related content on social media means that there is a lack of evidence upon which to base relevant practice and policy changes. This is particularly relevant at the moment in a number of countries, including Australia, where there is debate regarding better regulation of social media platforms, and a review of the Online Safety Act is underway [14, 15]. We conducted a large-scale national survey to help to address this gap.

## Methods

### Study aims

Study aims were to examine the extent to which Australian young people (aged 15–25) and adults (aged 26 and above): (i) are exposed to self-harm- and suicide-related content on social media; (ii) use social media to create content about self-harm and suicide; and (iii) use social media to seek support for self-harm and suicide. We also examined: (iv) the perceived impact of these activities on wellbeing; and (v) respondents’ views on the safety features implemented by social media companies, as they relate to self-harm- and suicide-related content. Young people were categorised as those aged 15 to 25 years. We chose an upper limit of 25 years to align with criteria used by youth mental health services in Australia (e.g., Orygen and Headspace) and we selected 15 years as our lower limit to align with other national youth surveys in Australia (e.g., Mission Australia, Australian Institute of Health and Welfare).

### Study design and setting

A national cross-sectional survey was conducted. The study was led by researchers at Orygen and the University of Melbourne. The fieldwork was conducted by research company, Roy Morgan Research (RMR; https://www.roymorgan.com/*).*

### Participants

#### Sampling

The study aimed to recruit a sample of 3,530 individuals from across Australia using quota sampling. Recruitment quotas were set with respect to age, gender, and area in accordance with the Australian Bureau of Statistics population estimates (ABS, 2023). This included 3,000 individuals aged 15 and above recruited from the Roy Morgan Single Source Panel, plus an additional 530 to increase the numbers of young people (*n* = 280), young people who identified as lesbian, gay, bisexual, transgender, queer/questioning or asexual (LGBTQIA+; *n* = 125), and young migrants (*n* = 125), for the purpose of two related PhD projects (hereafter referred to as ‘boost populations’). The boost populations were recruited from an additional panel managed by Pureprofile and snowball sampling whereby parents or guardians from the Roy Morgan panel were invited to nominate young people aged 15 to 17. Separate quotas were adopted for the boost populations without specific reference to gender or location.

The target sample size of 3,530 was considered sufficient to answer the research questions.

#### Recruitment

Recruitment was conducted via the following steps. First, an email or SMS was sent to eligible members of the RMR panel. Panel members were identified by their demographic characteristics which allowed RMR to fill the various quotas. Second, the survey was released to the Pureprofile panel. Third, snowballing was used to increase the number of people aged under 18 via parents or guardians.

Because it can be hard to recruit young people, a $20 gift card was offered to respondents aged 15 to 25 as an incentive.

### Survey instrument

The survey was adapted from the one used by The Samaritans (see above) (https://media.samaritans.org/documents/Samaritans_How_social_media_users_experience_self-harm_and_suicide_content_WEB_v3.pdf) in partnership with two youth advisers. It included:

- Demographic information;
- Social media experiences, including exposure to self-harm- and suicide-related content, the creation of self-harm- and suicide-related content, using social media to seek support for self-harm and/or suicide, and the impact of engaging with online self-harm- and suicide-related content;
- Views on the safety features employed by platforms;
- Wellbeing assessed by the K10, a 10-item validated measure of psychological distress [27];
- Previous experience of self-harm or suicide, including suicide bereavement. The following definition of self-harm was included in the survey: “Self-harm is defined as an act in which a person injures or poisons themselves with a motive that may or may not involve the intention of ending their life and survives. This does not include misuse of alcohol and other substances or body image problems and disordered eating patterns”.
- Those who endorsed previous self-harm or suicidal thoughts or behaviours were asked a series of questions about their experiences of, and reasons for, communicating on social media about self-harm and/or suicide and how this made them feel.
- A final set of questions asked participants about their beliefs about suicide and will be reported separately.

The survey primarily comprised predetermined response options (with response options including ‘yes’, ‘no’, ‘prefer not to answer’) and a series of 5-point Likert scales. Skip logic and branching were used to tailor the flow of the survey based on respondents’ answers. The survey included approximately 50 questions; however, those who had not engaged with self-harm- or suicide-related content on social media completed a briefer version compared to those who had.

This study adopted a two-step approach to ascertaining gender. Participants were asked to identify their sex at birth and select their gender identity from a list of 15 options (or describe a different term). Gender was then categorised based on alignment or difference between participants’ reported gender identity and birth-assigned sex. Participants for whom there was alignment between birth-assigned sex and gender were coded as cisgender, and those whose gender identity was different from their birth-assigned sex were coded as trans and gender diverse.

### Data collection

Data collection was conducted by RMR between January and March 2024. The survey was administered online, and all data were collected and stored on the RMR server, using the Forsta survey platform (https://www.forsta.com/resources/forsta-surveys/*).*

#### Pilot study

The survey was pilot tested in December 2023 with 52 participants from the RMR panel. Aims were to test the programming, structure and duration of the survey, and to ensure that it flowed smoothly, the skip logic was working, and respondents could complete it easily. Data analysis indicated that the survey routing worked as intended, and that most respondents understood and were willing to answer the questions. The average interview length of the pilot survey was 17.9 min, and no changes were required.

### Data cleaning and analysis

Because the survey was administered online and skip logic was used, there was minimal missing data. However, to address the risk of duplicate or fraudulent responses the following strategies were implemented. First, a ‘speeder check’ was conducted to ensure participants did not complete the survey too quickly. Second, a ‘straight-line’ check was conducted to ensure participants did not consistently select the same response on multiple consecutive questions. Because of the survey structure, if a participant selected ‘prefer not to answer’ over 10 times it was considered a case of ‘straight-lining’ and the record was flagged as potentially poor quality. Third, a ‘duplicate check’ was implemented. This involved examining responses from the same IP address and cross-checking variables such as age and gender. If a respondent submitted two responses, the second submission was removed. If a respondent raised three quality check flags the record was removed. Quality checking was conducted throughout data collection so that progress and quota size could be monitored.

#### Data analysis

Statistical analysis was conducted using R, version 4.3.3. Frequencies and percentages were calculated for categorical variables and the mean and standard deviation were calculated for continuous variables. A series of univariate and multivariate logistic regressions were conducted to compare young people (aged between 15 and 25 years) and adults (aged 26 years and older) on outcomes of interest (exposure to self-harm and/or suicide content online, searching for self-harm and/or suicide content online, creating self-harm and/or suicide content and seeking support on social media as outcome variables). Multivariate models controlled for gender (woman/man), personal lived experience (yes/no), family or friend with lived experience including suicide bereavement (yes/no), Aboriginal and/or Torres Strait Islander (yes/no), LGBTQIA+ (yes/no), migrant (yes/no) and location (metropolitan/regional). Whilst gender identity was coded as binary (man/woman) our analysis also included transgender men and women. Subsequent univariate and multivariate logistic regressions were conducted to compare young people with a personal lived experience of suicidal ideation, self-harm or suicide to those without on outcomes of interest (as stated above).

‘Prefer not to answer’ responses were treated as missing data and were not included in models. All models were created using the ‘glm’ function in R which handles missing data using listwise deletion.

### Ethics and safety

The study received approval from the Melbourne University Human Research Ethics Committee on 27/10/2023 (Ref: 2023-27352-46587-3). All participants were required to provide written consent and parental consent was required for those under 18.

Helpline information was included at the beginning and end of the survey. In addition, an interactive icon was placed on every page of the survey and when respondents tapped or hovered their cursor over this icon, helpline information appeared.

## Results

Results are presented below for the whole sample, and for young people (i.e., those aged 25 and under) and adults (i.e., those aged 26 and over).

### Sample and sample characteristics

In total 8,464 individuals commenced the survey; 2,790 from the RMR panel, 784 from the Pureprofile panel and 159 from snowballing. Of these 1,161 did not complete it, 74 were deemed ineligible (screened out) and 188 were excluded due to poor quality data. A further 3,492 responses were not used because quotas for that group were full. This left a final sample of 3,549; 895 young people aged 15 to 25 and 2,654 people aged 26 and upwards. The mean age of the overall sample was 43.6 (Young people Mage = 21.1, Adult Mage = 51.2). Just over half (50.9%, *n* = 1,805) identified as cisgender women and 45.5% (*n* = 1,613) as cisgender men.

Psychological distress scores ranged from 10 to 50. Scores were higher for young people compared to adults (24.0 versus 18.7) indicating mild levels of psychological distress. Just over thirty-five per cent of the sample (*n* = 1,264; 44.4% of young people and 32.7% of adults) reported personal lived experience of suicidal ideation, self-harm or suicide and 36% (*n* = 1279) reported having a friend or family member with lived experience (37.9% of young people and 35.4% of adults). Just over half of those with personal lived experience (*n* = 650, 51.4%) also reported having a friend or family member with lived experience. See Table 1.

**Table 1:** Demographic characteristics

	Entire sample (*n* = 3,549) % (*n*)	Young people aged ≤ 25 (*n* = 895) % (*n*)	Adults aged > 25 (*n* = 2,654) % (*n*)
Demographic variables			
Age			
Range	15–96	15–25	26–96
Mean (SD)	43.6 (19.4)	21.1 (3.2)	51.2 (16.5)
Gender			
Cisgender Woman	50.9 (1,805)	52.2 (467)	50.4 (1,338)
Cisgender Man	45.5 (1,613),	42.3 (379)	46.5 (1,234)
Trans and Gender Diverse	2.6 (91)	4.2 (38)	2.0 (53)
Don’t know or questioning, prefer not to say, other	1.1 (40)	1.2 (11)	1.1 (29)
Sexual Orientation			
Straight (heterosexual)	82.3 (2,920)	72.7 (651)	85. 5 (2,269)
LGBQA+	15.7 (556)	23.6 (211)	13.0 (345)
Don’t know or questioning, prefer not to say, other	2.1 (73)	3.7 (33)	1.5 (40)
Birth Country			
Australia	72.5 (2,573)	83.0 (743)	69.0 (1,830)
Another Country	27.5 (976)	17.0 (152)	31.0 (824)
Aboriginal or Torres Strait Islander			
Aboriginal	2.5 (89)	4.6 (41)	1.8 (48)
Torres Strait Islander	0.2 (6)	0.3 (3)	0.1 (3)
Aboriginal and Torres Strait Islander	0.2 (6)	0.2 (2)	0.2 (4)
Prefer not to answer	0.9 (33)	0.8 (7)	1.0 (26)
Location			
Metropolitan	66.6 (2,364)	71.2 (637)	65.1 (1,727)
Regional	33.4 (1,185)	28.8 (258)	34.9 (927)
Primary language spoken at home			
English	92.1 (3,270)	88.8 (795)	93.3 (2,475)
Language other than English	7.7 (274)	11.1 (99)	6.6 (175)
Prefer not to say	0.1 (5)	0.1 (1)	0.2 (4)
Lived experience			
Lived experience of either suicidal ideation, self-harm or suicide	35.6 (1,264)	44.4 (397)	32.7 (867)
Family or friend with lived experience of either suicidal ideation, self-harm or suicide	36.0 (1,279)	37.9 (339)	35.4 (940)
Clinical variables			
K10	20.0 (8.6)	24.0 (9.0)	18.7 (8.0)

Lived experience of either suicidal ideation, self-harm or suicide = ‘Yes, I have self-harmed’ and/or ‘Yes, I have attempted suicide’ and/or ‘Yes, I have had suicidal thoughts (also called suicidal ideation)’. Family or friend with lived experience of either suicidal ideation, self-harm or suicide = ‘Yes, someone important to me has self-harmed or attempted suicide’ and/or ‘Yes, I have been bereaved by suicide’

### Exposure to self-harm- and suicide-related content online

**Fig. 1:**
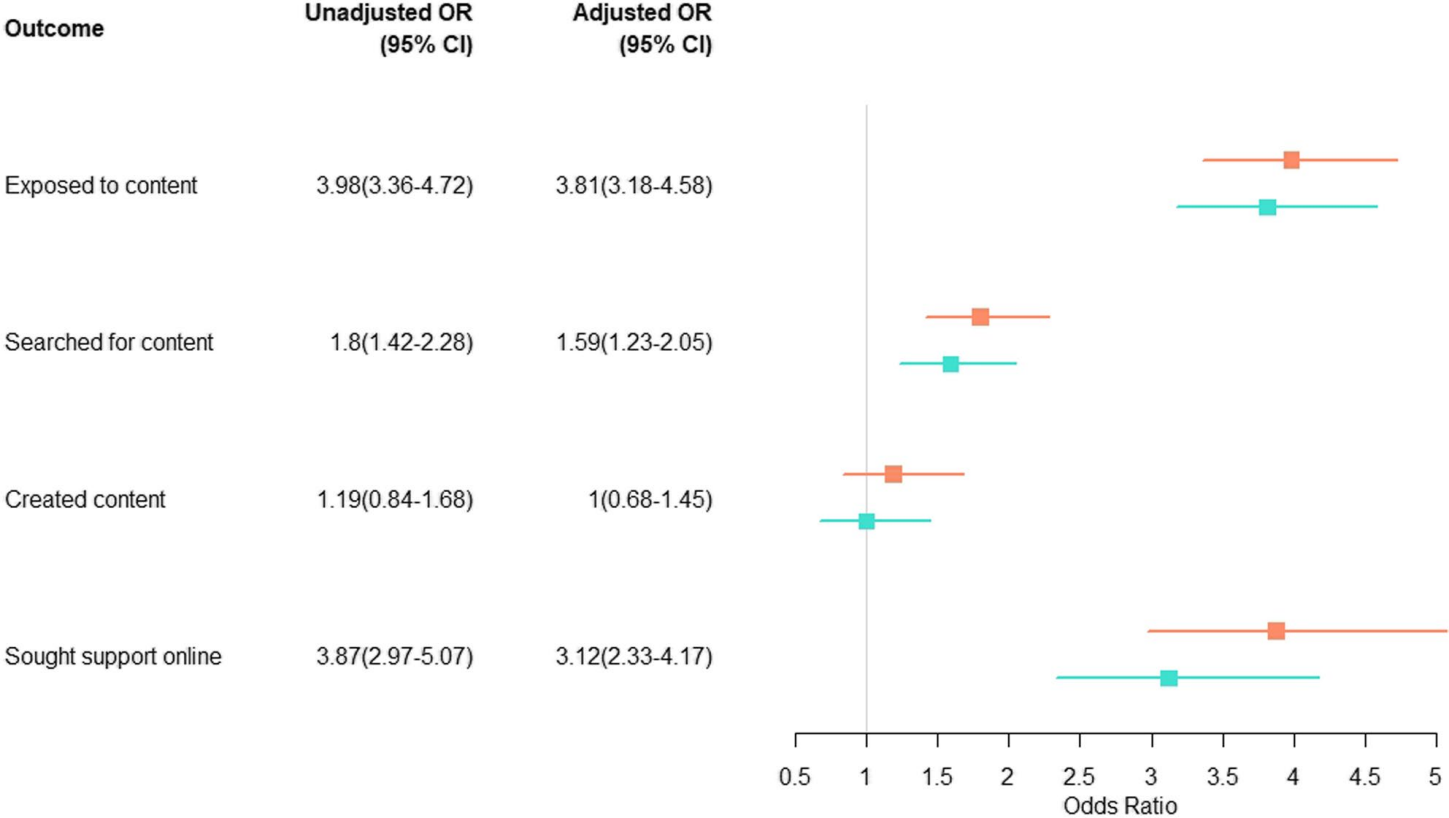
Unadjusted and Adjusted OR and 95% CI for young people compared to adults on outcomes of interest

Just over half of the overall sample had been exposed to self-harm- or suicide-related content online (51.6%; *n* = 1,831). Of them, 21.4% were under the age of 14 at the time of first exposure; a further 23.0% were aged between 14 and 16. The odds of exposure were almost four times higher among young people compared to adults (Adjusted OR 3.81; 95%CI: 3.18–4.58). Young people with a lived experience of suicidal ideation, self-harm and/or suicide compared to those without had greater odds of exposure (Adjusted OR = 2.65; 95%CI: 1.77–4.04). Of the exposed group, 19.7% (*n* = 360) had actively searched for this content; this represents 10.1% of the total sample. As above, the odds of actively searching were greater among young people compared to adults (see Fig. 1) and among young people with lived experience compared to those without (Adjusted OR = 7.13; 95%CI: 4.37–12.04).

#### Impact of exposure to online self-harm- and suicide-related content

Respondents who reported having been exposed to self-harm- and suicide-related content were asked a series of questions about the impact this had on them. Of the 1,831 participants who were asked this question, 63.3% (*n* = 1159) reported that it worsened their mood whilst 3.2% (*n* = 59) reported that it improved their mood. Almost thirty per cent (*n* = 528) reported that it neither improved nor worsened their mood and 4.6% (*n* = 85) said they ‘didn’t know’.

Respondents who had endorsed engaging in self-harm (*n* = 556) were asked if they had engaged in self-harm as a result of the content they had seen; 16.9% (*n* = 91) said they had done so, with the odds being over four times greater for young people than adults (Adjusted OR 4.02, 95%CI: 2.42–6.86). Almost three quarters (69.2%, *n* = 63) of respondents who had self-harmed as a result of seeing content online (adults and young people) reported doing so in the same or similar manner as depicted online.

### Creating self-harm and suicide-related content online

Of the entire sample, 4.1% (*n* = 145) had posted or created content about self-harm or suicide.

Respondents (*n* = 145) were then asked to rate the impact creating this content had on them using a series of Likert scales, ranging from one to five. Approximately one-third (33.8%; *n* = 49) reported finding this helpful; 32.4% (*n* = 47) reported that it stopped them self-harming and 25.5% (*n* = 37) reported that it stopped them feeling suicidal. In contrast, 22.1% (*n* = 32) reported finding it harmful, 26.2% (*n* = 38) reported that it increased their desire to self-harm and 26.2% (*n* = 38) reported that it increased their suicidal ideation.

### Seeking support online

Respondents with either their own lived experience of self-harm or suicide, or experience of supporting someone else, were asked if they had ever used social media to seek support for self-harm or suicide and if so why. Of the 1,893 people asked this question, 14.1% (*n* = 267) endorsed having done so. The odds were three times greater among young people compared to adults (Adjusted OR 3.12, 95% CI 2.33–4.17; See Fig. 1). The reasons cited for seeking support online are shown in Table 2. The most common reasons given were to connect with others with similar experiences, because it provided free and easy-to-access support, and because it allowed people to seek help without feeling like a burden to others.

**Table 2:** The reasons people sought support on social media

	Entire sample (*n* = 267) % (*n*)	Young people (*n* = 141) % (*n*)	Adults (*n* = 126) % (*n*)
Reasons given			
Connect with others with similar experiences	41.2 (110)	42.6 (60)	39.7 (50)
Free and easy to access information and/or support	44.9 (120)	51.8 (73)	37.3 (47)
In order not to worry or burden friends and family	43.1 (115)	47.5 (67)	38.1 (48)
Could turn to my online friends in times of need	37.8 (101)	37.6 (53)	38.1 (48)
Distract myself from self-harm or suicide	37.5 (100)	35.5 (50)	39.7 (50)
Found a sense of belonging and connection on social media	32.2% (86)	35.5 (50)	28.6% (36)
Learn coping strategies	32.6 (87)	34.0 (48)	31.0 (39)
Found people online who cared about me	33.0 (88)	36.2 (51)	29.4 (37)
No one I could talk to in-person	33.7 (90)	35.5 (50)	31.7 (40)
Stigma-free environment	30.7 (82)	32.6 (46)	28.6 (36)
Not able to access professional support	19.9 (53)	19.1 (27)	20.6% (26)
Access support from people outside of my cultural group	18.4 (49)	18.4 (26)	18.3 (23)
Access LGBTQA + affirming support	17.6 (47)	21.3 (30)	13.5 (17)
Access culturally appropriate support	14.2 (38)	14.9 (21)	13.5 (17)

### Respondents’ views on safety features used by social media platforms

Finally, all respondents who had been exposed to self-harm- or suicide-related content (*n* = 1,831) were asked their views about a range of safety features used by social media companies. Here respondents were able to select multiple responses. As can be seen in Table 3, the most commonly endorsed features were including content warnings ahead of posts about self-harm or suicide, promoting educational content, and including helpline information in posts about self-harm or suicide. Young people compared to adults had greater odds of suggesting social media companies should implement content warnings (Adjusted OR1.60, 95%CI:1.30–1.97). Young people compared to adults did not have greater odds of suggesting social media companies should include helpline information or promote educational content.

**Table 3:** Respondents’ views on the safety features used by social media companies

	Entire sample (*n* = 1,831) % (*n*)	Young people (*n* = 675) % (*n*)	Adults (*n* = 1,156) % (*n*)
Strategies			
Include content warnings	59.3 (1,085)	67.4 (455)	54.5 (630)
Promote educational content	58.9 (1,078)	58.2 (393)	59.3 (685)
Include helpline information	56.1 (1,028)	58.5 (395)	54.8 (633)
Redirect search results to helpful educational content	52.2 (956)	56.3 (380)	49.8 (576)
Remove or censor any images depicting self-harm or suicide	45.5 (834)	50.7 (342)	42.6 (492)
Include in-platform safety planning functions	40.4 (739)	43.1 (291)	38.8 (448)
Redirect search results to helplines	40.8 (747)	44.0 (297)	38.9 (450)
Prevent from appearing in suggested content	40.3 (738)	45.2 (305)	37.5 (433)
Nominate a contact person in suicidal crisis	35.7 (653)	33.6 (227)	36.9 (426)
Shadow banning self-harm/suicide content (i.e., reducing its visibility)	32.3 (592)	38.2 (258)	28.9 (334)
Use artificial intelligence to identify users at risk and send links to support	29.2 (534)	31.0 (209)	28.1 (325)
Restrict accounts repeatedly posting	25.1 (459)	31.3 (211)	21.5 (248)
None of the above	3.8 (70)	2.5 (17)	4.6 (53)

## Discussion

This paper reports findings from a national cross sectional survey study that examined Australians’ experiences of online communication about self-harm and suicide, and compared the experiences of young people to the experiences of adults. In general, young people were exposed to, and created, this type of content more than adults; this included using social media to seek support for self-harm or suicide.

Overall, around half of respondents had been exposed to self-harm- or suicide-related content online. As reported previously by the Samaritans (https://media.samaritans.org/documents/Samaritans_How_social_media_users_experience_self-harm_and_suicide_content_WEB_v3.pdf), in the current study, exposure was often accidental and in nearly half of cases, occurred before the age of 16. Rates of exposure were higher in young people compared to adults, and in young people with lived experience. For most people, exposure to self-harm- or suicide-related content worsened their mood; again, this echoed the Samaritans’ study (https://media.samaritans.org/documents/Samaritans_How_social_media_users_experience_self-harm_and_suicide_content_WEB_v3.pdf). Furthermore, an important minority of people reported that exposure led to further self-harm, and some of these reported engaging in similar methods of self-harm to those depicted online.

It is unsurprising that younger people and those with lived experience were more likely to be exposed to this type of content online, even accidentally, given that both groups were also more likely to actively search for it and create it themselves. Platform algorithms are programmed to deliver content that is tailored to the individual and their online behaviour [17], so if a person with a history of self-harm or suicidal ideation has sought help for these problems online, the platform algorithms are then more likely to suggest this type of content in the future, even when the person is not actively searching for it.

Social media has been available for a greater proportion of young people’s lives, and they spend more time on social media than adults [16]. They are confident users of social media and in the current study, they were also more likely to seek support on social media platforms for self-harm or suicide. Therefore, again it is unsurprising that they were more likely to be exposed to this type of content. However, given that in most people exposure worsened their mood, and for some it exacerbated their self-harm, steps to limit people’s exposure to this type of content should be taken. Such steps might include further limiting the volume of self-harm- or suicide-related content allowed on platforms, and adapting the algorithms to reduce the frequency with which it is delivered to users, even if they have previously searched for it. Steps to reduce the potential harms associated with exposure may also include ensuring that all self-harm- or suicide-related content is accompanied by educational and/or helpline information.

The fact that some individuals reported engaging in self-harm using similar methods to those depicted online gives weight to concerns that some types of content may lead to imitative behaviour [18]. The #chatsafe guidelines, developed to facilitate safe communication about self-harm and suicide on social media, recommend avoiding sharing content that portrays 1graphic images or details of self-harm or suicide methods for this reason [19, 20]. These guidelines have been widely disseminated via a series of social media campaigns [21], and have been shown to increase young people’s perceived ability to communicate safely online about suicide [22, 23]. However, given that many people are exposed to this content before the age of 16, their impact might be further strengthened if they are also supported by educational programs embedded in school curricula and supplemented with training programs for school staff, parents and carers. Indeed, our previous research has indicated strong support for this [13]. The Office of the e-Safety Commissioner in Australia has developed a suite of resources for young people, families and educators regarding online safety; however, none focus specifically on self-harm or suicide, and this could be a useful next step [24].

A significant proportion of our sample, in particular young people, reported using social media to seek support for self-harm or suicide, and this should not be overlooked. The main reasons given for seeking support were to connect with others, because it was free and easy to access and did not burden family or friends. Other reasons included finding a sense of belonging and connection online and because it was perceived to be less stigmatising. This echoes the findings of previous studies, reinforcing the fact that using social media is not always inherently harmful [11], and for some it may, in fact, reduce risk by reducing burdensomeness and increasing connectedness [25].

A quarter of respondents sought online support because they could not access it offline. It is well documented that access to mental health services is limited with long waitlists and people frequently turned away, even when presenting with self-harm and suicide risk [26]. Given the rising rates of help-seeking for self-harm and suicide among young people [6] there is a clear need for additional resources for clinical services (and other supports) in order that demand for help can be met. However, in the absence of this, there are questions about the potentially important role being played by social media for some people. This includes how we can better capitalise on the features of social media to deliver evidence-based support to those who need it and how we better integrate on-and off-line support to provide young people with a more accessible and seamless experience.

Finally, the study examined respondents’ views on the safety features currently employed by social media platforms. The most commonly endorsed features were the use of content warnings, the promotion of educational resources and the inclusion of helpline information. Each of these features was found more helpful by young people than adults and by those with lived experience. The academic literature reports conflicting findings regarding the use of content warnings [26], and in our recent Delphi study, no consensus was reached about their utility [19]. However, in the current study, both young people and those with lived experience reported finding them useful, which suggests that they may be beneficial for those that need them most. New features developed by platforms should reflect the evolving ways that young people use social media and should be done so in consultation with young people with lived experience using ‘safety by design’ principles [24].

### Strengths and limitations

The key strength of this study is that it is the first to quantify, in a large national sample, the extent to which Australians are exposed to, and create, self-harm- and suicide-related content on social media and the impact this has on them, and how this differs between young people and adults. It also examined the extent to which social media is used to seek support for self-harm and suicide and, unlike many previous studies, it distinguished between passive exposure to content (which appears to be mostly harmful) and active posting or help-seeking (which appears to be helpful).

However, the study was not without limitations. First, the use of quota sampling and the boosted sample means that the findings cannot be generalised to the population at large. Second, as with all surveys of this nature it was based on self-report data and therefore subject to recall bias. In addition, there were some variables where the numbers were too small for statistical testing and some demographic variables were collapsed to provide greater statistical power and for ease of reporting. For example, ‘lived experience’ included people with their own lived experience of self-harm or suicidal thoughts or behaviours plus people who had supported others. It was beyond the study scope to examine the impact of different volumes of exposure to certain types of content, the precise nature of the self-harm- or suicide-related content people were exposed to, or the extent to which different types of content were perceived to be helpful or harmful (e.g., graphic images of self-harm compared to evidence-based treatment information). That said, our studies testing the #chatsafe campaigns have shown them to be safe and acceptable to young people, which suggests that self-harm- or suicide-related content that is educational and preventative in nature may be safe to deliver [22, 23].

## Conclusion

There is significant debate among policy makers and in the mainstream media about the need for greater regulation and age restrictions on access to social media platforms, with some Australian politicians calling for a blanket social media ban for users under the age of 16. Indeed, our findings that many individuals are exposed to self-harm and suicide-related content under the age of 16, combined with the fact that the majority of those exposed to it found it harmful, certainly support the need for changes to current practice. However, many young people also sought support online for important reasons, including lack of access to help offline, suggesting that this avenue for support should not be removed altogether, and certainly should not be removed until it can be replaced with a safe alternative. Rather, a more nuanced, collaborative and evidence-based approach is required.

Findings from the current study indicate that changing the ways in which platform algorithms currently operate, delivering educational programs for younger users (plus families and educators) and policy responses that distinguish between the needs of young people and adults, and which reflect the day-to-day reality of young people’s lives may all be beneficial.

## Supplementary Information

Supplementary Material 1.

## Data Availability

The following will be made available to researchers upon receipt of an approved and methodologically sound proposal: individual participant data after de-identification, plus the analytic code that underlie the results reported in this article (text, tables and figures). The study protocol and consent forms will also be made available. Proposals should be directed to jo.robinson@orygen.org.au to request access. Upon approval, data requesters will be required to sign a data access agreement. Proposals may be submitted beginning 9 months and ending 36 months following article publication.
